# Detecting disease rare alleles using single SNPs in families and haplotyping in unrelated subjects from the Genetic Analysis Workshop 17 data

**DOI:** 10.1186/1753-6561-5-S9-S96

**Published:** 2011-11-29

**Authors:** Aldi T Kraja, Jacek Czajkowski, Mary F Feitosa, Ingrid B Borecki, Michael A Province

**Affiliations:** 1 Division of Statistical Genomics, Center for Genome Sciences & Systems Biology, Washington University School of Medicine, 4444 Forest Park Boulevard, St. Louis, MO 63110, USA

## Abstract

We present an evaluation of discovery power for two association tests that work well with common alleles but are applied to the Genetic Analysis Workshop 17 simulations with rare causative single-nucleotide polymorphisms (SNPs) (minor allele frequency [MAF] < 1%). The methods used were genome-wide single-SNP association tests based on a linear mixed-effects model for discovery and applied to the familial sample and sliding windows haplotype association tests for replication, implemented within causative genes in the unrelated individuals sample. Both methods are evaluated with respect to the simulated trait Q2. The linear mixed-effects model and haplotype association tests failed to detect the rare alleles of the simulated associations. In contrast, the linear mixed-effects model and haplotype association tests detected effects for the most important simulated SNPs with MAF > 1%. We conclude that these findings reflect inadequate statistical power (the result of small simulated samples) for the complex genetic model that underlies these data.

## Background

Based on evolutionary history, it is expected that human genomes will be arranged into local regions with high haplotype similarity intermingled with local regions with haplotype diversity. In the diverse haplotype regions, rare alleles may play an important role in creating the foundation for individual subjects’ susceptibility or resistance to a particular disease. It is a common practice in genetic research to identify causative disease regions in the framework of association using single single-nucleotide polymorphism (SNP) tests or by grouping neighboring regions under identifiable haplotypes and to test their association with disease and quantitative traits. Genetic Analysis Workshop 17 (GAW17) provides 200 replicates of simulated data of a family-based cohort with eight large families and 200 replicates of simulated data for unrelated individuals. This simulated problem is challenging, because both sets of data are relatively small (*n* = 697). We investigate the two problems independently to determine whether the family-based association tests have power to detect rare allele effects and whether rare allele effects in the simulated genes can also be detected by haplotype analysis of the unrelated individuals sample.

## Methods

The GAW17 data represent 200 replicates of simulated phenotypes for a sample of 697 subjects organized in 8 large families (referred in this article as the familial sample) and 200 replicates of simulated phenotypes for a separate sample of data of 697 unrelated individuals (referred to here as the unrelated sample). Genotypes from the 1000 Genomes Project were used as the genotype sample for the unrelated sample. The GAW17 simulation authors [1] used the family data set, by means of the program CHRSIM [2], to drop the phased founder genotypes throughout the rest of the pedigree by considering a single obligate crossover event occurring on each chromosome. The same two genotype sets were used for all 200 phenotypic simulation replicates for the familial or unrelated sample.

We analyzed the unrelated sample genotypes for linkage disequilibrium using HaploView software (version 4.2), with the purpose of identifying tag SNPs [3]. The options we used in a batch mode run of HaploView for identifying tag SNPs were –pairwiseTagging and –tagrsqcutoff 0.8. We used the number of discovered tag SNPs as a denominator for extrapolating the Bonferroni genome-wide significance threshold for a single-SNP association test (see Results section).

After setting a genome-wide significance threshold, we applied a linear mixed effects (LME) model to the familial sample. The LME statistical analyses are based on linear estimates of additive genetic effects of single SNPs. The LME model is:(1)

where *β*_1_*g* measures the change in *Y* as a result of the additive change in the genotype *g*∈(−1, 0, +1) where −1 is the code for the homozygote genotype for the minor allele, 0 is the code for the heterozygote, and +1 is the code for the homozygote for the major allele. We implemented association tests using a statistical mixed model on the familial sample via the mixed procedure (PROC MIXED) of SAS (version 9.2, Linux OS). The “repeated” statement was used with sub=pedid, which defines the structure of the *R* matrix, and the covariance structure was selected as UN. We tested all 24,487 SNPs included in the simulation, although we had prior knowledge of the GAW17 simulation answers. With such prior knowledge we focused on trait Q2.

Q2 was simulated as a quantitative trait, influenced primarily by 72 SNPs in 13 genes, with 1–15 functional variants per gene and with minor allele frequencies (MAFs) ranging from 0.07% to 17.07%. The residual heritability of Q2 was simulated to be 29%. Most of the genes affecting the Q2 trait were selected to be related to cardiovascular disease risk and inflammation, and they are located on chromosomes 2, 3, 6–12, and 17. Before the LME association tests, we performed a stepwise regression for Q2 within Sex to remove the effects of Age and Age^2^. As a result, we produced a Q2 residual, which we then used as the dependent variable *Y* in our analyses. In the statistical analyses, the variable Sex was included as a covariate (*β*_2_cov).

We applied haplotype association tests only in the unrelated individuals sample and specifically within causative simulated genes. We viewed the haplotype analysis as a replication analysis by completing it in the unrelated sample for only trait Q2 simulated causative genes. This was justified by the preceding gain of information from the LME analysis and from the knowledge we had of the simulated model for Q2. We implemented the haplotype analyses using Haplo.Stats, version 1.4.4 [4]. The haplotypes studied were created by sliding windows of two and windows of three adjacent SNPs. This program was run in a batch mode in R software, version 2.10.0, for Linux OS. The Haplo.Stats package includes an EM algorithm to estimate haplotype frequencies for unrelated subjects. In the haplo.score function (within this package), Schaid et al. [4] implemented the score statistics *U_β_* and *V_β_*, where:(2)

is a measure of covariation of residuals with the recoded genotypes and:(3)

is the variance. To test the association of the trait and marker haplotypes, Schaid et al. [4] also provided a global score statistic *S*,(4)

with a chi-square distribution and degrees of freedom equal to the rank of *V_β_*.

Finally, we use two definitions to summarize the LME results: sensitivity and specificity. Sensitivity is the proportion of true-positive causative SNPs that have a significant test result in 200 replications; and specificity is the proportion of true-negative SNPs that have a negative test result in 200 replications. Both sensitivity and specificity are expressed as percentages.

## Results

**Figure 1:**
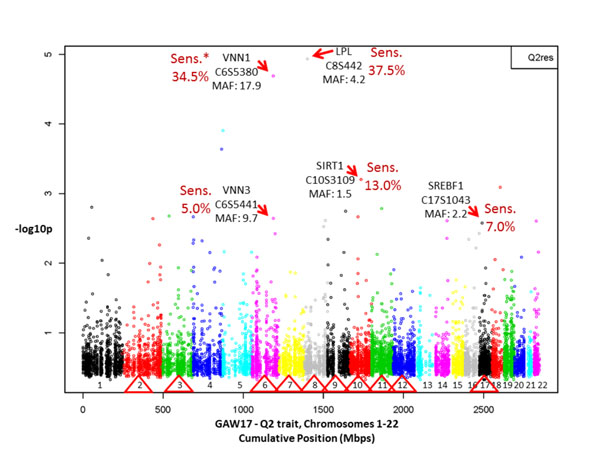
**Genome-wide linear mixed effects additive model association test results of 200 replications of family-based cohort data on Q2 residuals**. None of the SNPs’ −log_10_*p* mean values passed the 5.4 genome-wide threshold. Sens. = sensitivity. Red triangles mark chromosomes that were simulated with Q2 causative SNPs (72 of them in 13 genes).

The HaploView linkage disequilibrium analysis on all 24,487 markers in the unrelated sample identified 11,626 tag SNPs. Therefore our Bonferroni genome-wide significance (*α* = 0.05) threshold *p*-value for independent tests was 4.3 × 10^−6^ (−log_10_*p* = 5.4). Figure 1 shows the results of the average −log_10_*p* single-SNP genome-wide significance of 200 replications in families produced by fitting an additive genetic model on Q2 residuals. The average of −log_10_*p* for 200 replications was for all simulated SNPs under the 5.4 (−log_10_*p*) threshold. In particular, a group of SNPs simulated in genes *VNN1* (C6S5380, MAF = 17.9%) on chromosome 6 and *LPL* (C8S442, MAF = 4.2%) showed to some extent significant results.

**Figure 2:**
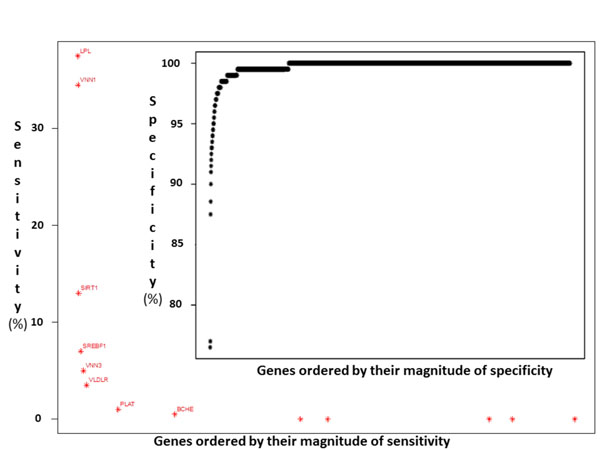
Sensitivity and specificity of SNPs discovery by using the linear mixed-effects additive model on gene bases for the Q2 simulated trait

Figure 2 shows the sensitivity per simulated gene by selecting the most significant representative SNP. For example, sensitivity was 37.5% for *LPL* (marker C8S442) and 34.5% for *VNN1* (marker C6S5380). The specificity for noncausative genes of these tests was very high, >99% (Figure 2).

**Figure 3:**
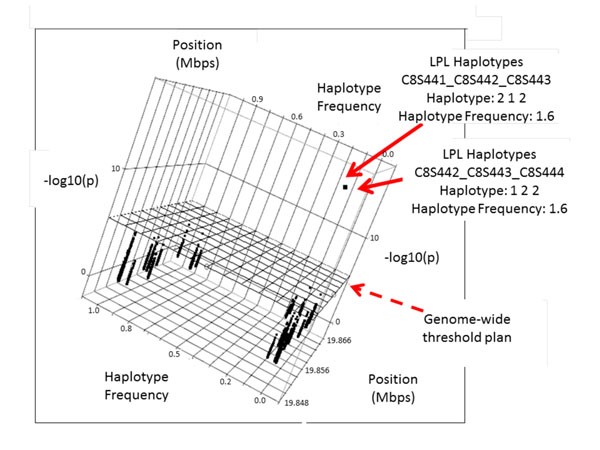
**Three-marker sliding windows haplotypes for the *LPL* gene**. Haplotypes containing the SNP C8S442, although close to rare haplotypes (frequency 1.6), showed distinct association with Q2 trait.

**Figure 4:**
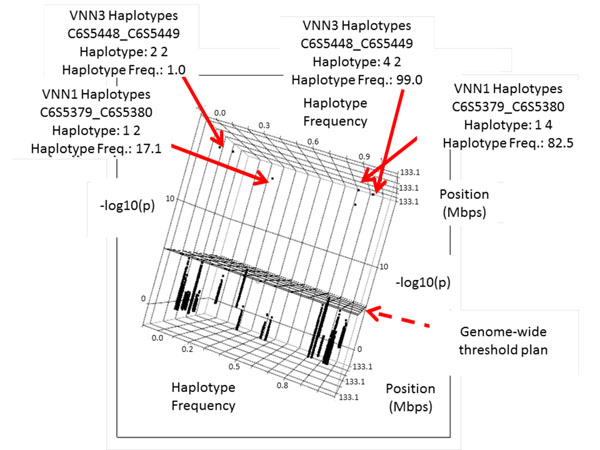
**Two-marker sliding windows haplotypes for genes *VNN3* and *VNN1.*** Haplotypes containing C6S5380 and C6S5449 show distinct association with Q2.

We performed association tests for all causative genes, with sliding windows of haplotypes taken two or three markers at a time, in the unrelated sample. Both types of sliding windows incorporated the detectable effects of the simulated data. For example, haplotype C8S441-C8S442-C8S443 with a frequency of 1.6% in the gene *LPL* was associated significantly with Q2 (Figure 3). Another haplotype that included marker C8S442 (C8S442-C8S443-C8S444) and had the same frequency of 1.6% also showed significant association with Q2. In genes *VNN3* and *VNN1* a few haplotypes (with two-marker windows) showed significant association with Q2, for example, haplotype C6S5448-C6S5449 with a frequency of 1% and haplotype C6S5379-C6S5380 with a frequency of 17.1% (Figure 4). When using the genome-wide significance threshold of −log_10_*p* of 5.4, sensitivity on genes *VNN3* and *VNN1* of the most significant haplotype (C6S5379-C6S5380) was 11.5%, and for *LPL* (haplotype C8S441-C8S442-C8443) the sensitivity was 2%. Otherwise, if we considered the haplotype analysis in the unrelated sample as a replication of the preliminary discovery in the familial sample and set the significance threshold to a value corresponding to the number of haplotype windows tests per gene, then the corresponding sensitivity became 22.9% for *LPL* and 64.5% for *VNN3* and *VNN1*. Other haplotypes from other rare allele SNP simulated regions did not confirm significant results.

## Discussion

Detecting association of very rare alleles (MAF < 1%) with disease or quantitative traits in familial data of small sample size is challenging. One would have guessed that in the GAW17 data set, because of its simulation as a transmission of selected alleles from the unrelated sample into the familial sample, one or more rare alleles would have been transmitted as common alleles in the familial sample. In the simulated data such conversion from rare alleles into common ones with the existing small sample sizes (from unrelated founders into familial data) was not present. Therefore in both sets of genotypic data, simulations were performed mostly on the very rare alleles in 96% of the 72 selected SNPs for Q2. The average −log_10_*p*-value of genome-wide association tests on the simulated data was less than the significance threshold. This illustrates the necessity of finding other methods that manage association tests for rare alleles.

With the massive data dumps coming from new sequencing technologies, rare alleles will be captured more often in the data and will be the aim of many studies. Moreover, it is believed that rare alleles are the gold dust that confer the rest of disease heritability. It is possible that, in contrast to this simulation, sample sizes in real data will become larger as the costs of sequencing decrease. But in the existing case we needed to find ways to increase our statistical power to identify the rare causative variants in association with disease or quantitative traits. Therefore we used a two-step analysis design: the LME model for discovering genome-wide causative SNPs or genes in the familial sample and haplotype association tests for replicating primary findings, but now in the unrelated sample.

The haplotype association test has more power to identify the SNPs’ effect on disease and quantitative traits. The haplotype association tests produced significant results for several common haplotypes, including those with a frequency close to 1%. However, this method also failed to identify any other significant haplotypes in association with Q2 from the very rare simulated SNPs (MAF < 1%). Based on our results for 200 replications, we conclude that failing to detect the very rare causative SNPs was not a failure of the methods but more a reflection of the low statistical power available with the small sample size of the simulated data.

## Conclusions

The genotypic data analyzed from the 1000 Genomes Project (sampled real data) based on unrelated individuals show that for the selected gene regions there is an important saving of about 53% when tag SNPs are used rather than the full sequence. The other dimension of the data, the sample size of 697 individuals used in this simulation, was small, and thus although sequence data were available, the power of association analysis was low. Therefore the additive single-SNP association tests based on LME models in small samples of data failed to detect the simulated causative very rare polymorphisms. The association tests based on haplotype analyses improved the *p*-value strength by replicating discovered causative polymorphisms, but they still failed with very rare alleles, reflecting the inadequate statistical power of small sample sizes in the simulated data.

## Competing interests

The authors declare that there are no competing interests.

## Authors’ contributions

ATK and JC carried out the analyses; ATK, JC, MFF, IBB, and MAP planned the analyses, wrote the manuscript as well as revised it. All authors read and approved the final manuscript.
